# Implementation of Fractal Dimension and Self-Organizing Map to Detect Toxic Effects of Toluene on Movement Tracks of* Daphnia magna*

**DOI:** 10.1155/2018/2637209

**Published:** 2018-02-26

**Authors:** Yuedan Liu, Chunlei Xia, Zhongya Fan, Renren Wu, Xianglin Chen, Zuoyi Liu

**Affiliations:** ^1^The Key Laboratory of Water and Air Pollution Control of Guangdong Province, South China Institute of Environmental Sciences, MEP, Guangzhou 510655, China; ^2^Yantai Institute of Coastal Zone Research, Chinese Academy of Sciences, Yantai 264003, China

## Abstract

Movement behaviors of an indicator species,* Daphnia magna*, in response to contaminants have been implemented to monitor environmental disturbances. Complexity in movement tracks of* Daphnia magna* was characterized by use of fractal dimension and self-organizing map. The individual movement tracks of* D. magna* were continuously recorded for 24 hours before and after treatments with toluene at the concentration of 10 mg/L, respectively. The general complexity in movement tracks (10 minutes) was characterized by fractal dimension. Results showed that average fractal dimension of movement tracks was decreased from 1.62 to 1.22 after treatments. The instantaneous movement parameters of movement segments in 5 s were input into the self-organizing map to investigate the swimming pattern changes under stresses of toluene. Abnormal behaviors of* D. magna* are more frequently observed after treatments than before treatments. Computational methods in ecological informatics could be utilized to obtain the useful information in behavioral data of* D. magna* and would be further applied as an in situ monitoring tool in water environment.

## 1. Introduction

Behavior is an organism response to environmental stimuli defined as an action, a reaction, or a function of a system under a specific circumstance [1]. Thus, behavioral response is supposed to be an outward projection of central networks of physiology pertaining to the individuals. Automatic detection of behavioral responses of water species has been applied as an efficient tool for biomonitoring in aquatic environment, because animal behaviors would be suitable as indicators for various pollutants [2, 3]. A large number of behavioral studies on chemical effects at low concentrations have been reported subjected to various taxa including insects [4], crustaceans [5], nematodes [6], snails [7], and fish [8]. The information involved in animal movement behavior, however, has been regarded as difficult to exact due to complexity residing in the data, and so effective methods to analyze behaviors without a priori knowledge are important in the behavioral monitoring [9].

Motion segmentation would be useful to provide available information for computational analysis of animal movement tracks. The purpose of segmentation is to make complex behaviors easy to analyze [10]. Motion segmentation was widely applied based on the computer vision technique [11, 12]. The movement tracks of water indicator species could be accordingly segmented and patterned to detect the abnormal behaviors occurring when individuals are exposed to chemicals [13, 14]. Movement segmentation and movement patterning, however, have never been used to comparatively study animal behavioral data for different time scales.

Fractal dimension is generally used to characterize fractal patterns by featuring the complexity as a ratio of the change in detail to the change proportionally [15]. Fractal dimensions are fractional numbers originating from filling of space, in which fractal number of area is integer 2 and that of volume is integer 3. Fractal dimensions are commonly applied to determine the self-similarity properties and natural structural patterns. Recently, fractal dimension has been used to describe behavioral patterns of animals such as fruit flies [16] and chironomids [17]. Self-organizing map (SOM) is an artificial neural network that is trained through unsupervised learning to obtain a low-dimensional and discretized description of the input data and is therefore a process to conduct dimensionality reduction [18]. SOM has primarily been used in analysis of complex behaviors in response to the extreme environmental stresses in ecological risk assessment, because it can efficiently identify the patterns of animal responses based on behavioral parameters [19, 20]. SOM was also applied to differentiate animal behaviors with various genotypes in response to environmental pollutants [4, 16]. In addition, behavioral classification with the SOM has been expanded for use of clinical applications [21]. Thus, the two methods, fractal dimension and SOM, have a prospective application to study the movement behaviors of indicator species exposed to chemical stresses and further to detect the existence of pollutants in the water.

The zooplankton,* Daphnia magna*, is a fresh or brackish water organism widely used as a standard indicator species in a variety of ecological studies.* D. magna* has numerous advantages as an experimental organism. This Cladoceran species is relatively easy to keep in the laboratory, has a fast generation time, and can be maintained at high population densities in limited storage [22]. Moreover, the body transparency of* D. magna* helps in observations of its inner structure in response to chemical treatments on anatomy, while its behavioral sensitivity to a broad range of chemical stressors helps for investigation on environmental monitoring [22, 23].

This study is aimed at (1) detecting chemical effects of toluene on movement behaviors of* Daphnia magna* based on general shape and movement patterns of swimming tracks after treatments of toluene in static water environment and (2) verifying the fact that computational analyses (i.e., fractal dimension and SOM) could be used to effectively reveal the behavioral information of* D. magna* for longer and shorter time units. As an important component of petroleum hydrocarbons, toluene has been reported to be toxic to water species, because it can produce negative effects on growth and reproductive performance of aquatic animals [24]. The individual movement tracks of* D. magna* before and after the treatments of toluene were obtained through an image processing system. Subsequently, the movement tracks were segmented into a longer time unit (i.e., 10 minutes) and were further cut into a relatively shorter time unit (i.e., 5 seconds). The movement parameters of the two time units were input, respectively, analyzed by two computational methods, by fractal dimension to extract general movement complexity from longer tracks and by SOM to investigate movement pattern from shorter tracks.

## 2. Materials and Methods

### 2.1. Indicator Species and Test Chemical

*Daphnia magna* (1 day young) used in the experiment was cultured according to the standard rearing procedure [25]. The young population of* D. magna* was hatched at the South China Institute of Environmental Sciences, China, and stocked in the laboratory with temperature at 25 ± 2°C, pH at 7.0 ± 0.3 (average ± standard deviation), and photophase for 14 hours with illumination from 3000–4500 lx and scotophase for 10 hours. Toluene (Sigma-Aldrich Co.) at a concentration of 10 mg/L was added to the water in a confined observation cage, because the 24 h LC50 or EC50 of toluene on* D. magna* was reported as 53–500 mg/L according to the previous studies [26, 27]. Ten healthy individuals of* D. magna* were randomly selected and individually transferred to a nontoxic acrid cage (60 mm × 50 mm × 10 mm) filled with filtered water that was moved from the stock tank. The individuals of* D. magna* were acclimated to the observation environments for a half hour and then were individually vertically recorded continuously for 24 hours before the treatments and after the treatments, respectively. Food and oxygen were not supplied to the system during the entire observation to minimize the amount of noise data.

### 2.2. Behavioral Observation System

**Figure 1:**
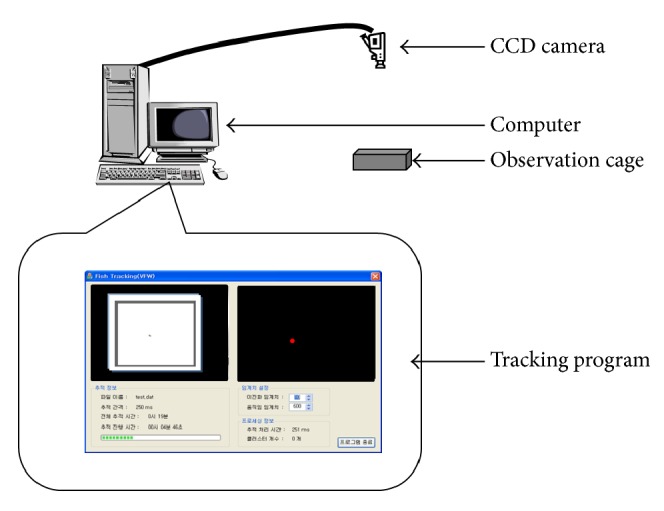
Behavior observation system for* Daphnia magna.*

The individual movement tracks of* D. magna* were recorded and recognized by a computer vision system with a CCD camera (Hitachi KP-D 20 BU®), an acrid observation cage, a timer, an Analog/Digital interface card (Matrox Morphis®), and image recognition software (0.25 s/frame) (Figure 1). A robust background subtraction algorithm based on frame differencing with filter was used to image recognition in this study [28]. The snapshot was sent to the system to recognize coordinates of target individuals in the spatial and time domains. The interval of 0.25 s segment was considered as sufficiently short in presenting information of movement behavioral data in detail, while the response time for 24 hours is generally enough to observe chemical effects for acute toxicological treatment [29]. Meanwhile, the time frame was also suitable in observing the behaviors of test organisms not only at the open but also at the boundary area [30]. Some response behaviors due to toxic effects such as compulsion and trembling might be expressed less than 0.25 s, but this behavior in extremely short duration was not considered in the present study.

### 2.3. Calculation of Movement Parameters

In order to characterize activities and shapes of instantaneous movement tracks in response to chemical treatments, movement parameters were automatically calculated based on individual locations in each frame by the behavioral observation system. Based on preliminary studies [29, 30], the following 7 parameters were screened to characterize the movement data for each 5 s segment: speed (mm/s), acceleration (mm/s^2^), locomotory rate (mm/s), stop number (*n*), stop time (s), turning rate (rad/s, angular change divide by time), and meander (rad/mm, angle change per movement distance). Speed (movement distance divided by the observation time), acceleration (speed difference divided by time), and stop time (total duration without movement) represent the general linear activity of the test organisms. The locomotory rate was additionally measured to show how fast the test organisms move. The speed indicates the average movement distance during the total observation time, while the locomotory rate is the average movement distance when the organisms move, excluding the total duration of the stop time. The turning rate (angular change divide by time) and meander (rad/mm, angular change divided by distance) were used to reflect the turning behavior of the test organisms.

### 2.4. Fractal Dimension

The general complexity information in the behaviors of* D. magna* was extracted from the images of movement tracks using fractal dimension [15].

(1) $$\begin{array}{c} D=\underset{r\to 0}{\lim}\frac{\log\left(N\left(r\right)\right)}{\log\left(1/r\right)}, \end{array}$$

where *N*(*r*) is the least number of boxes of length *r* with points (positions of individuals) and *r* is the size of boxes that need to completely cover the object. Given a binary image of *M* × *M* pixels, where *M* is a power of 2, fractal dimension could be transferred to

(2) $$\begin{array}{c} D\approx \frac{\log\left(N\left(\delta \right)\right)}{\log\left(1/\delta \right)}, \end{array}$$

where *δ* is box size, *δ* = *rM*  (0 < *r* < 1). The calculation procedure could be conducted by three steps as follows. (1) Set of box sizes *δ* for laying grids on the image of movement tracks by using a sampling method is generated [31]. Each grid becomes a box of size *δ* × *δ*. (2) For each *δ*, the number of boxes *N*(*δ*) containing positions of daphnia completely is counted. (3) Fractal dimension *D* is obtained from the slope of points (log⁡(1/*δ*), log⁡(*N*(*δ*))). Based on the definition of topological dimension, while the dimension value of a line is 1.0 and that of a surface is 2.0, the number of fractal dimensions would be any value from 1.0 to 2.0, since the movements of* D. magna* were tracked in two dimensions by the observation system. The duration of the movement tracks was set to 10 minutes, which was properly enough to show response effects based upon the movement behaviors of other species such as fruit flies [16] and chironomids [17].

### 2.5. Self-Organizing Map

The data matrix including 7 parameters of 1000 movement tracks (5 s), before and after treatments, respectively, was randomly selected and input to train the self-organizing map (SOM) [18]. The Euclidian distance (*d*_*j*_(*t*)) for the *j*th node between weight at iteration time *t* and the input vector was trained following the processes:

(3) $$\begin{array}{c} d_{j}\left(\;t\;\right)=\overset{P-1}{\underset{i=0}{\sum }}{\left[\;x_{i}-w_{ij}\left(\;t\;\right)\;\right]}^{2}, \end{array}$$

where *x* is input vector of the *i*th variable, *w*_*ij*_(*t*) is weight between the *i*th variable and the *j*th node, and *P* is the number of the parameter.

The best-matching neuron with the minimum distance was selected as the winner. The weight vectors between the inputs and the nodes were calculated, when the data were input to the networks.

(4) $$\begin{array}{c} w_{ij}\left(\;t+1\;\right)=w_{ij}\left(\;t\;\right)+a\left(\;t\;\right)\left[\;x\left(\;t\;\right)-w_{ij}\left(\;t\;\right)\;\right], \end{array}$$

where *t* is iteration time and *α*(*t*) is training rate. The weights of the best-matching unit and its close neurons were updated towards the input vector through an interactive calculation in the lattice. Consequently, the similarity between the movement segments is reflected on the output SOM map. Ward's linkage method was used to reveal the degree of association between the movement data based on the dendrogram using the Euclidean distance matrix [32, 33]. The learning process of the SOM was conducted by the SOM Toolbox (The Mathworks, R2011) [34].

### 2.6. Statistical Analysis

Paired-sample *t*-test was used to test the significance of difference for the movement parameters, fractal dimension, and amount of movement patterns before and after treatments of toluene. SPSS 15.0 was used for statistical analysis [35].

## 3. Results

### 3.1. Behavioral Activity

**Figure 2:**
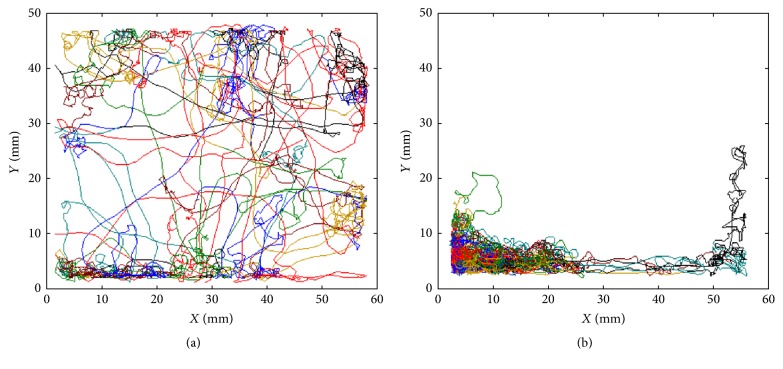
Movement tracks of* Daphnia magna *treated with toluene at 10 mg/L. (a) Before treatment and (b) after treatment. Colors on tracks indicate movement segments in 10 s time interval.

The general activity of* D. magna* can be clearly seen from the recorded movement tracks before and after the treatments of toluene, respectively (Figure 2). In general, the individuals of* D. magna* were usually more active before the treatments. The movement tracks spanned a large area of the observation cage with smooth and linear shapes with individual variations in activity (Figure 2(a)). The shaking or zig-zag segments was rarely observed in the movement tracks. On the contrary, typical tracks with more abnormal irregular swirls or turns were showed after the treatments under the chemical effects (Figure 2(b)). The degree of activity decreased and swimming range was reduced accordingly. The irregular turns in the movement tracks indicate that behaviors of observed individuals were severely affected by the chemical treatment. The toxic effects of toluene on observed individuals were also presented with changes in movement parameters. For example, speed decreased from 5.14 ± 0.98 mm/s to 3.47 ± 0.85 mm/s (one-tailed paired-sample *t*-test, *t* = 7.72, DF = 9, *p* < 0.01), while turning rate increased from 1.33 ± 0.51 rad/s to 3.73 ± 1.19 rad/s (one-tailed paired-sample *t*-test, *t* = −13.52, DF = 9, *p* < 0.01) after the treatments.

### 3.2. Behavioral Complexity

**Figure 3:**
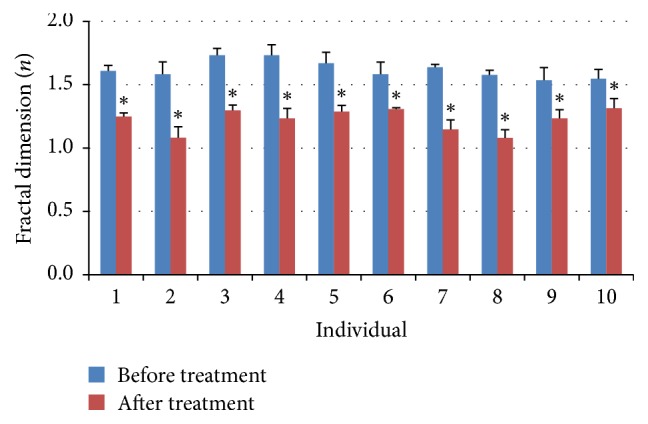
Fractal dimension of movement tracks (10 minutes) before and after treatments of observed individuals. *∗* indicates significant difference, *p* < 0.01.

Behavioral complexity in movement tracks of* D. magna* was subsequently detected by the fractal dimension in two dimensions (Figure 3). Fractal dimension of the movement tracks being close to the maximum value 2 means that the individual searches and passes by all the area evenly. Reversely, the fractal dimension less than 2 indicates that daphnia chooses movement patterns in a certain position. After the treatments, fractal dimension of movement tracks of individuals in 10 minutes was significantly decreased from 1.62 ± 0.24 to 1.22 ± 0.21 (one-tailed paired-sample *t*-test, *t* = 6.68, DF = 9, *p* < 0.01). The results overall indicated decrease in complexity of movement data after treatments.

### 3.3. Movement Patterns

**Figure 4:**
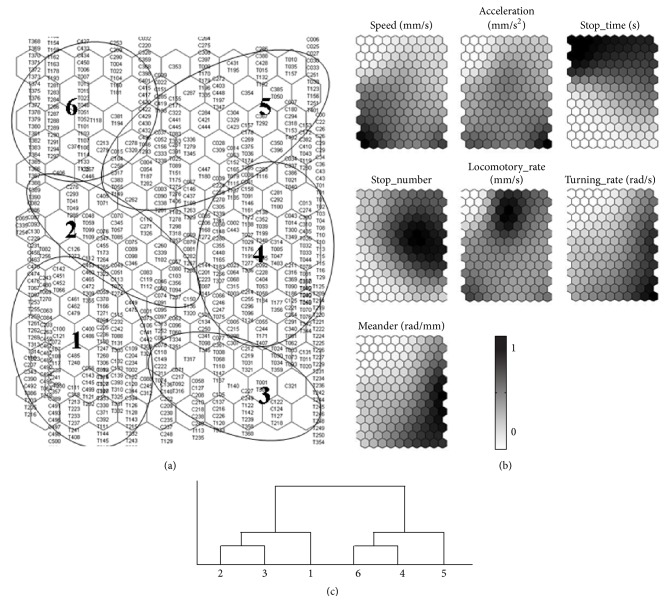
The map trained by using the SOM for pattering movement segments of* D. magna* in 5 s. (a) Six clusters classified by the SOM (“C” represents movement segments before treatments, while “T” stands for movement segments after treatments). (b) Profile of the parameters matching the clusters based on the trained SOM. The values in the vertical bar in the top row indicate normalized parameters. (c) Dendrogram according to Ward's linkage method.

**Figure 5:**
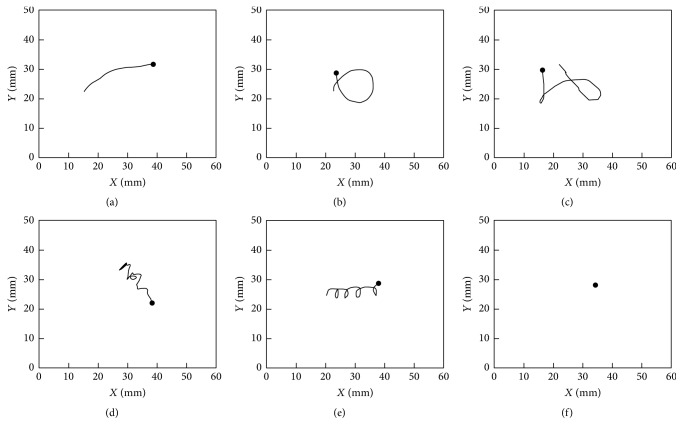
Diagrammatic sketch of movement patterns in 5 s segments based on the trained SOM (a) line (P1); (b) loop (P2); (c) cross (P3); (d) shaking (P4); (e) swirl (P5); and (f) stay (P6).

Movement segments (5 s) were patterned by use of the SOM with seven parameters calculated from the observation data. The movement tracks were accordingly grouped on the SOM before and after the treatments, respectively (Figure 4(a)). The movement segments before treatments were dominated by the marks “C” in the bottom of SOM map, while the segments marked with “T” were located in the top in a majority after treatments. Six typical movement patterns were defined and identified according to the linkage clustering. The clusters accordingly were defined as the following movement patterns: (1) line (Figure 5(a), P1 in cluster 1), long forward step, appeared to be smooth and linear; (2) loop (Figure 5(b), P2 in cluster 2) was shorter than p1 in one direction with smooth shape and fast speed; (3) cross (Figure 5(c), P3 in cluster 3), was characterized by combination of p1 and p2; (4) shaking (Figure 5(d), P4 in cluster 4), was alternatively left and right turning with spanning a small area; (5) swirl (Figure 5(e), P5 in cluster 5) was small circular shape keeping a clockwise or anticlockwise direction; and (6) stay (Figure 5(f), P6 in cluster 6) was to be a movement pattern with the lowest speed.

The profiles of the parameters in 5 s segments were visualized based on the grouped SOM units (Figure 4(b)). The distance between clusters was provided based upon the weights or thresholds of closeness in dendrogram (Figure 4(c)). The movement pattern P1 was primarily featured by linear movements with the highest speed, the shortest stop time, and the smallest stop number. P2 indicated a loop characterized with high speed, relatively high locomotory rate, and middle acceleration. P3 presented the highest acceleration, turning rate, and the low stop time. The shaking pattern P4 at the middle right of the map was characterized as relatively low speed and acceleration, high stop number, and turning rate, while swirl in P5 showed parameters with long stop time, low speed, and acceleration compared with P4. Remarkably being different from other patterns, P6 stay was the most nonactive movement with the greatest level of stop time, stop number, and the lowest speed and acceleration.

### 3.4. Proportion of Movement Patterns

The compositions of movement patterns in 1000 segments, before and after, respectively, were summarized as shown in Figure 5. In general, the percentages of active movement patterns (P1, P2, and P3) were significantly increased, while the proportions of inactive movement patterns were universally decreased under the chemical stresses. The dominant patterns were P1 and P2; in particular, p1 holds 25.4% of the total number of segments. The continuous movements (P3) and stay (P6) were also dominant before the treatments, while P4 and P5 occurred with minimal frequency.

The proportion of the movement patterns was significantly changed by the chemical effects after the treatments. The percentage of active movement patterns (i.e., linear or continuous direction in movement sequence, P1, P2 and P3) decreased, while those of nonactive movement patterns (i.e., zig-zag type movements, P4 and P5, or stay, P6) increased after the chemical treatments. It was notable that the proportion of linear movement (P1) decreased distinctively from 25.4% to 17.1% after the treatments. The stop pattern (P6), however, substantially increased from 14.7% to 22.5%. The change in pattern frequency indicated that the chemical effects were accordingly projected onto the spatial-time domain of the movement data. The paired-sample *t*-test accordingly showed statistical significance in each pattern at *p* < 0.01 (*t* values: 30.56 (P1), 21.45 (P2), 22.38 (P3), −24.42 (P4), −27.58 (P5), and −26.91 (P6)) before and after the treatments.

## 4. Discussion

Automatic monitoring based on behavioral data of indicator species has attracted much attention in risk assessment in water ecosystems, because the monitoring methods could fill the gap between macroscale (e.g., community structure) and microscale (e.g., molecular response) measurements. Detection of stressor with early signals through behavioral changes of indicator organisms is ecologically more relevant, faster, and cheaper than chemical detection [36]. Compared to survival as an endpoint, behavioral parameters have been proven to be often between 10–100 times more sensitive to the chemicals [1, 37]. Once a determined behavior can be quantified, it has the potential to be used as a biomarker in the assessment of stress [38]. A remarkable advantage of behavioral monitoring is that any human process is not required during the observation period. However, behavioral data are considered to be difficult to analyze, because numerous biological factors are involved in a complex manner. With the rapid development of computer computational ability of personal computer, a longer term automatic real-time monitoring becomes true through the behavioral observation system, including data collection, data analysis, and decision making for early warning, which is nearly impossible by human process. Behavioral monitoring could be conducted on the real-time basis without much demanding observation efforts and facilities; however, the application of the automatic monitoring system in the natural water environment is easily affected by many factors (e.g., individual variance, environmental conditions), and this problem should be solved by improving system robustness step by step in the future.

Considering that location density information is embedded in fractal dimension, the fractal dimension could be naturally used to present the activity information in animal movement behaviors (Figure 2). The complexity involved in movement behaviors reflected by the change of fractal dimension was accordingly elucidated in differentiating effects of internal and external stimuli even in two-dimensional tracks. Obviously, it would be more useful in quantifying the movement data if higher dimension could serve to reveal more complex behaviors. In this study, we applied two-dimensional data as an initial step of the study, and fractal dimension would be more suitable in presenting diverse behavioral changes if three-dimensional observations are used in the future.

**Figure 6:**
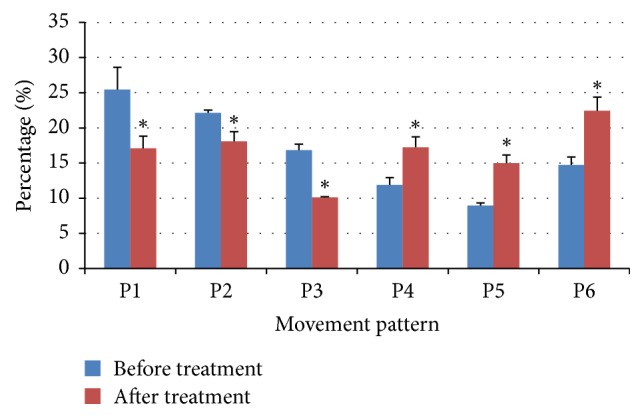
Percentage of movement patterns before and after treatments with toluene at 10 mg/L. *∗* indicates significant difference, *p* < 0.01.

Because of the complexity residing in a huge amount of data in two-dimensional movement, finding an efficient method to determine the pattern changes in movement behavior with conventional methods was not easy. Notably, the SOM process provides useful information on movement patterns regarding this type of complex behavioral data. Movement segments were clearly clustered on the output SOM map based upon inputting movement parameters (Figure 4), and the movement patterns were clearly differentiated to represent normal and abnormal behaviors (Figure 5). The proportion of linear or long step movement segment (p1, p2, and p3) decreased, while those of zig-zag movements (p4 and p5) or stay (p6) increased after the chemical treatments (Figure 6). Illustrations of changes in the movement patterns will be helpful for further characterizing the movement mechanisms of indicator species.

SOM was reported to be superior to conventional feature extraction methods such as empirical orthogonal functions or principal components analysis, with many advantages [39]. This is because the nonlinear SOM could minimize Euclidian distance between learned pattern vectors and data vectors and preserve the data topology rather than the variance. Through the training process, weights (i.e., movement parameters) of all neighborhoods are pushed to the same direction and similar items (i.e., movement segments) tend to occupy adjacent neurons. Therefore, SOM forms a trustable visualized map where similar samples are clustered close together and dissimilar ones apart. For the present study, through SOM analysis, the movement patterns of* D. magna* could be clustered into a certain number of groups that are maximally close to the “real patterns” under a specific circumstance. Thus, the differences in the movement tracks of* D. magna* were efficiently revealed before and after the treatments of toluene. Complex behavioral data could be extracted through the SOM and could be accordingly patterned to illustrate the overall view of behavioral response to chemical stressors. The SOM further suggested that objective characterization of complex behavioral data by the computational methods could effectively serve as the real-time and online monitoring tools.

Most movement parameters used in the present study mainly concern the instantaneous locomotion, while fractal dimension represents general movement complexity. We would like to focus on how the test organisms behave instantaneously and continuously in relatively short distances in a confined cage (Figure 1). Considering for practical use for monitoring in situ, small size arena would be more feasible. Since the individuals are reared in a limited area in the arena, the individuals occasionally stayed near the boundary area. Behaviors near the boundary area could be different from the movements of the individuals in the middle of the arena. In this study, however, a relatively low proportion of the observation time was spent near the boundary area. Considering that the boundary area could be determined as 2 mm inside the boundary [40], the specimens usually stayed less than 5% of the total observation time on average in the boundary area. In the future, however, a definition of behavioral states near boundary area should be checked further, because behavioral states in the boundary area might be characterized differently [41].

## 5. Conclusion

The toxic effects of toluene on* D. magna* could be expressed by the increase number of abnormal behaviors in the movement tracks for different time scales, for 10 minutes and 5 s long in the present study, respectively. The general complexity in movement tracks was clearly characterized by fractal dimension, while instantaneous movement patterns could efficiently extracted by SOM. Computational analysis on movement behaviors of* D. magna* could be an alternative monitoring tool to automatically detect chemicals in aquatic environment.
